# Power Generation by Reverse Electrodialysis in a Microfluidic Device with a Nafion Ion-Selective Membrane

**DOI:** 10.3390/mi7110205

**Published:** 2016-11-10

**Authors:** Tsung-Chen Tsai, Chia-Wei Liu, Ruey-Jen Yang

**Affiliations:** Department of Engineering Science, National Cheng Kung University, Tainan 70101, Taiwan; tsungchen0329@gmail.com (T.-C.T.); N96044353@mail.ncku.edu.tw (C.-W.L.)

**Keywords:** reverse electrodialysis, salinity gradient power, Gibbs free energy, Nafion membrane, energy conversion

## Abstract

An energy conversion microchip consisting of two circular microchambers and a Nafion-filled microchannel is fabricated using standard micro-electro-mechanical systems (MEMS) techniques. When the chambers are filled with KCl solutions with different concentrations, the Nafion microchannel acts as a cation-selective membrane and results in the generation of electrical power through a reverse electrodialysis (RED) process. The current-potential characteristics of the Nafion membrane are investigated for devices with various microchannel lengths and electrolyte concentration ratios. It is shown that for a given voltage, the current and generated power increase with a reducing channel length due to a lower resistance. In addition, a maximum power density of 755 mW/m^2^ is obtained given an electrolyte concentration ratio of 2000:1 (unit is mM). The optimal device efficiency is found to be 36% given a channel length of 1 mm and a concentration ratio of 1000:1 (mM). Finally, no enhancement of the short circuit current is observed at higher concentration ratios.

## 1. Introduction

Most of today’s energy is produced from fossil fuels (e.g., coal, oil and natural gas). However, this has serious implications for the environment, including most notably, the emission of greenhouse gases (CO_2_, SO_2_ and NO_x_) and global warming. To address the problems of environmental pollution and climate change and to prevent future resource depletion, it is necessary to develop alternative green energy sources, such as salinity gradient energy [1,2,3], biomass conversion [4], wind power [5], solar energy [6], and others. Of these various technologies, salinity gradient energy is one of the most attractive since seawater accounts for almost 70% of the planet’s surface. In the salinity gradient method, power is generated from the Gibbs free energy produced during the mixing of seawater and fresh water. The method was first proposed by Weinstein and Leitz [7] and has an estimated theoretical output capacity of up to 1.4–2.6 TW [8]. Suda et al. [9] investigated the performance of a dialytic battery consisting of 59 compartments made with 29 ion-exchange membrane pairs, each with an effective area of 80 cm^2^ per sheet. The results showed that while the output power reduced over time, a power density of 259 mW/m^2^ was possible during the initial mixing of seawater and river water. Veerman et al. [10] showed that for a scaled-up reverse electrodialysis (RED) stack consisting of 50 cells with a size of 25 cm × 75 cm, a maximum power density of 930 mW/m^2^ could be achieved given an appropriate hydrodynamic design of the stack. 

Advances in micro-electro-mechanical systems (MEMS) techniques have facilitated device miniaturization in many applications [11], including biomedical implants, micro-sensors, micro-batteries and portable personal electronics [12]. Furthermore, when combined with nanofluidics technology, MEMS-based devices provide the means to generate energy not only from large-scale systems, but also from micro/nano-scale devices [13]. The problem of power generation by RED in ion-selective microchannels has attracted significant attention in the recent literature. The application of micro-RED systems can be used for some resource-limited settings while travelling outdoors and could also be used as a counterpart of a solar-driven satellite just in case of power insufficiency. Kim et al. [14] showed that for silica nanochannels with heights of 4, 26 and 80 nm, respectively, the ion selectivity increased with a reducing concentration gradient and channel height. Thus, the optimal performance (a power density of 7.7 W/m^2^ and an efficiency of 31%) was obtained in the device with a channel height of 4 mm. Kang et al. [15] investigated the RED effect in anodic alumina nanopores given various pore lengths, pore radii and electrolyte concentrations. Overall, the results showed that a power output density of 9.9 W/m^2^ could be achieved given a suitable specification of the engineering parameters. Chang and Yang [16] presented a theoretical model based on a modified Onsager reciprocal relation and the Poisson–Boltzmann model for predicting the electrokinetic energy conversion efficiency in short-length nanofluidic channels. It was shown that the results obtained from the proposed model were in good agreement with those of the Poisson–Nernst–Planck (PNP) model in the absence of concentration polarization effects at the reservoirs. In a later study by the same group (Chang and Yang [17]), it was shown that the electrokinetic energy conversion efficiency of ion-selective nanopores could be increased to more than 40% by imposing a hydrodynamic slip ratio greater than 0.7.

Guo et al. [18] presented a fully-abiotic single-pore nanofluidic energy-harvesting system, developed by chemically-etching the nanopores, which achieved a system with a maximum power output of 26 pW. Tandon et al. [19] investigated the electroosmotic mobility properties of ion-selective microfluidic devices fabricated from hydrophobic polymers and showed that a higher ion mobility resulted in a greater diffusion current and an improved power. Cao et al. [20] compared the RED performance of single ion-selective nanopores for three different electrolytes, namely KCl, NaCl and LiCl. The results confirmed that the electric power and energy conversion efficiency are both dependent on a well-matched charge selectivity and ionic composition. In particular, it was shown that for the considered nanopores, the highest diffusion coefficient and power generation (45 pW) were obtained using the KCl solution. Chang et al. [21] investigated the diffusion potential and power generation performance of a microchip containing a Nafion ion-selective membrane given the use of three different electrolyte solutions with pH values of 3.8, 5.6 and 10.3, respectively. The performance of the device was found to improve with an increasing pH value, with a diffusion potential of 135 mV and a power generation of 339 pW observed for the electrolyte with a pH value of 10.3.

The present study fabricates a simple energy conversion microchip consisting of two circular microchambers connected by a Nafion-filled microchannel. When the chambers are filled with KCl solutions with different concentrations, the Nafion microchannel acts as a cation-selective membrane, and hence, electrical power is generated as the result of RED. The current-potential (*I-V*) characteristics, power generation performance and electrokinetic conversion efficiency of the proposed device are systematically examined for various values of the microchannel length and electrolyte concentration ratio.

## 2. Material and Methods

**Figure 1:**
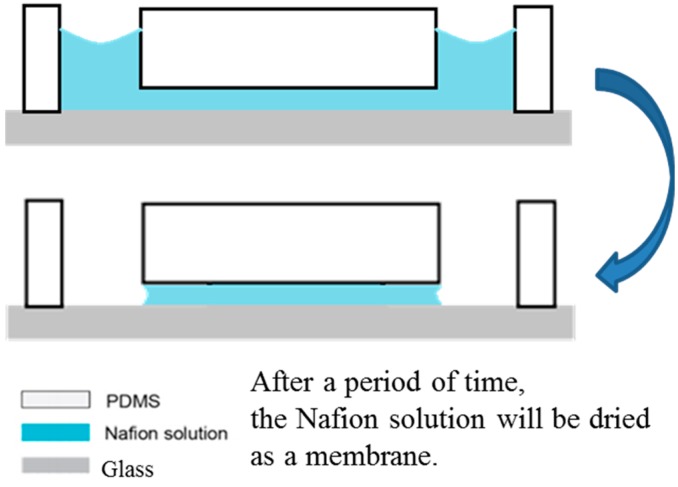
Fabrication of the Nafion ion-selective membrane in the microchannel.

A mold consisting of the two microchambers and the inter-connecting microchannel was patterned on a silicon wafer using standard MEMS techniques. A polydimethylsiloxane (PDMS) structure was then produced using a simple replication method. The PDMS structure was bonded to a blank glass substrate by an oxygen plasma treatment process in order to form the final RED chip. Nafion solution purchased from DuPont (DE-2021, Wilmington, DE, USA) was diluted with deionized (DI) water in a proportion of 5:1, then injected into one reservoir and allowed to fill the microchannel and the other reservoir under the effects of capillary forces (see Figure 1). The chip was then stored at room temperature for 8 h; causing the Nafion to solidify within the microchannel and reservoirs [22,23]. The solidified Nafion in the two reservoirs can be easily peeled off. The Nafion remaining in the microchannel acts as a cation-selective membrane and results in the generation of electrical power through a reverse electrodialysis (RED) process. 

**Figure 2:**
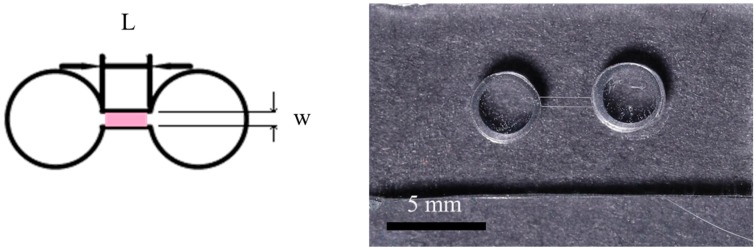
Basic configuration and photograph of the reverse electrodialysis (RED) device.

Figure 2 shows the basic configuration of the fabricated chip. Three devices were produced with microchannel lengths of 1, 2 and 3 mm, respectively. In every case, the microchannel had a height of 23 μm and a width of 500 μm. The dimensions of the fabricated microchannels were measured using ImageJ software and were found to deviate by no more than 13% from the corresponding design values. The RED experiments were performed using Ag/AgCl electrodes and KCl electrolyte solution with a pH value of 5.6 ± 0.2. Four different electrolyte concentration ratios (*C*_H_:*C*_L_) were considered, namely 10:1, 100:1, 1000:1 and 2000:1 (unit is mM). In each experiment, the potential and current were measured using a Keithley 2400 source meter (Keithley Instruments, Cleveland, OH, USA). Each case is conducted at least for four independent measurements.

## 3. Results and Discussion

**Figure 3:**
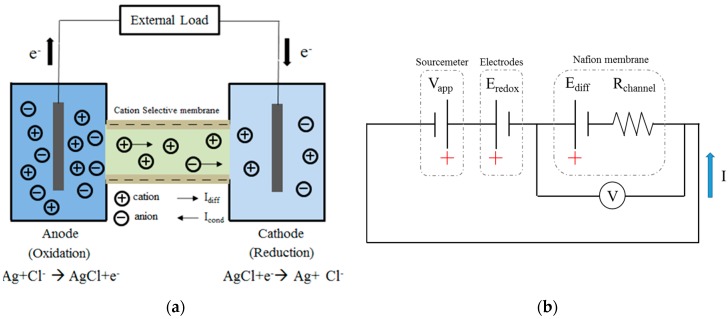
(**a**) Schematic illustration of the RED process in the cation-selective channel; and (**b**) the equivalent electrical circuit of the experimental setup.

Figure 3a shows a schematic illustration of the RED process in the proposed device. As shown, the left (anode) and right (cathode) reservoirs are filled with high- and low-concentration KCl solutions, respectively. As a consequence, a salinity gradient is formed along the length of the microchannel. Due to the ion-selectivity of the membrane, the cations and anions diffuse toward the low-concentration reservoir at different rates. Consequently, a difference in the positive and negative charges is produced at the two ends of the membrane. This difference results in the formation of a diffusion potential, which is subsequently converted into electrical energy through a process of reverse electrodialysis. Figure 3b shows the equivalent electrical circuit for the experimental setup used to evaluate the performance of the proposed RED device. Note that *V*_app_ is the potential applied by the source meter; *E*_redox_ is the potential produced by the redox reactions at the electrodes; *E*_diff_ is the diffusion potential; *R*_channel_ is the resistance of the microchannel filled with Nafion; and *I* is the output current generated by the microchip. The redox potential produced at different concentration ratios (*C*_H_:*C*_L_) can be expressed by the following Nernst relation [24,25]:

(1) $$E_{\mathrm{redox}}=\frac{RT}{zF}\ln\frac{\gamma_{C_{H}}C_{H}}{\gamma_{C_{L}}C_{L}}$$

where *R*, *T*, *z*, *F* and γ are the gas constant, absolute temperature, charge number, Faraday constant and mean activity coefficient, respectively. The diffusion potential can be expressed as [21]:

(2) $$E_{\mathrm{diff}}\;=\;\left({2t}_{+}\;-\;1\right)\frac{RT}{zF}\ln\frac{\gamma_{C_{H}}C_{H}}{\gamma_{C_{L}}C_{L}}$$

where $t_{+}$ is the transference number for the cations and can be expressed as *t*_+_ = *j*_+_/(*j*_+_ + *j*_−_), where *j*_+_ and *j*_−_ are the cation and anion fluxes, respectively. The transference number provides an index of the ion selectivity of the membrane. More specifically, *t*_+_ = 1 indicates that the membrane has full cation selectivity, while *t*_+_ = 0 indicates that the membrane has full anion selectivity.

When the system is connected to an external load, the output power is given by:

(3) $$V=E_{\mathrm{diff}}-IR_{\mathrm{channel}}=V_{\mathrm{app}}-E_{\mathrm{redox}}$$

(4) $$P_{\mathrm{out}}\;=\;IV\;=\;\frac{E_{\mathrm{diff}}^{2}R_{\mathrm{load}}}{{\left(R_{\mathrm{load}}+R_{\mathrm{channel}}\right)}^{2}}$$

The maximum output power is obtained when $R_{\mathrm{load}}\;=\;R_{\mathrm{channel}}$ and can be computed as [21,26]:

(5) $$P_{\max}=\frac{E_{\mathrm{diff}}^{2}}{{4R}_{\mathrm{channel}}}$$

**Figure 4:**
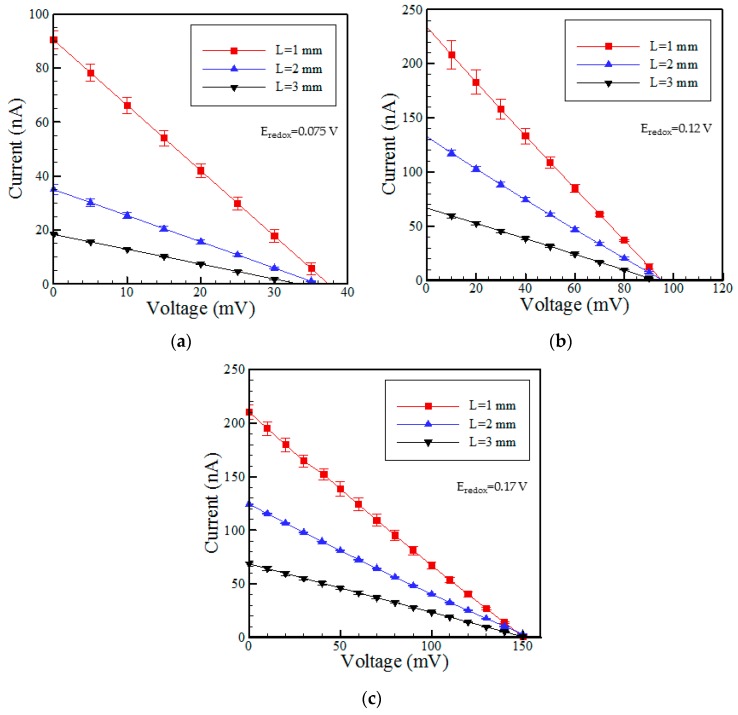
Current-potential curves for RED devices with different microchannel lengths and concentration ratios of: (**a**) 10:1; (**b**) 100:1; and (**c**) 1000:1 (unit is mM).

Figure 4a–c show the measured *I-V* curves of the three RED devices with different microchannel lengths given KCl concentration ratios of 10:1, 100:1 and 1000:1 (mM), respectively. It is seen that for all values of the concentration ratio, the channel resistance (*R* = *V*/*I*) reduces with a reducing microchannel length. As a result, the power generated by the RED device reduces as the microchannel length increases. 

**Figure 5:**
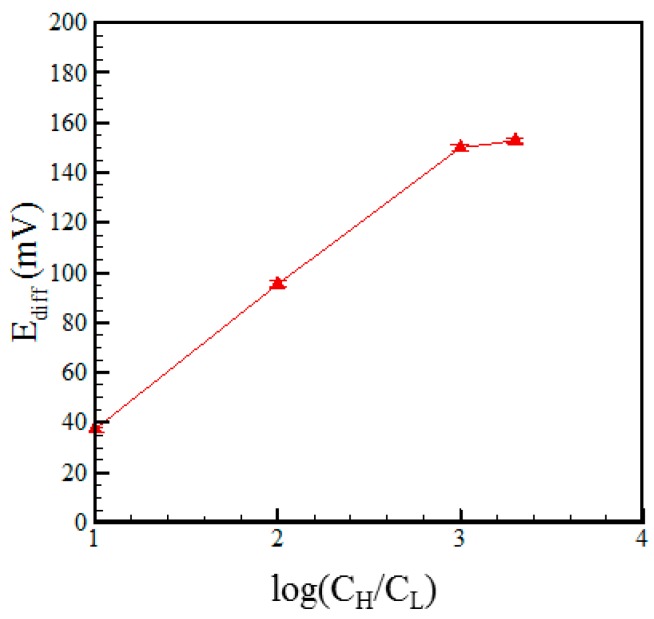
Variation of diffusion potential with concentration ratio given microchannel length of 1 mm.

**Figure 6:**
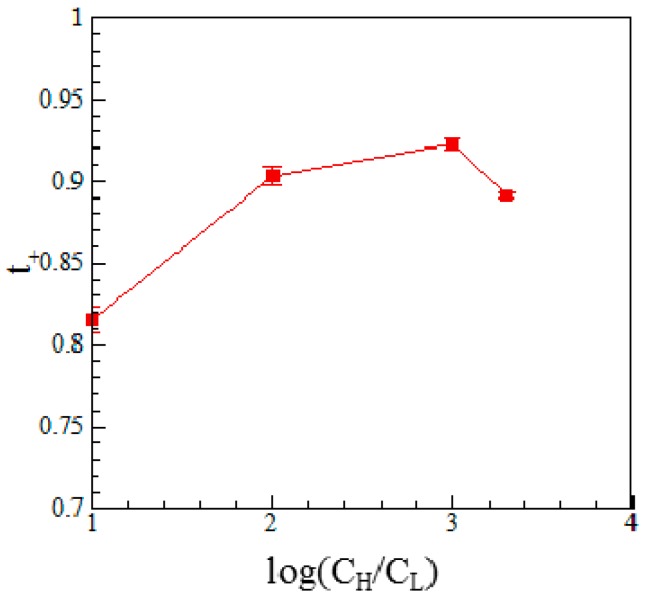
Variation of transference number with the concentration ratio given a microchannel length of 1 mm.

Figure 5 and Figure 6 show the variations of the diffusion potential and transference number, respectively, with the electrolyte concentration ratio given a microchannel length of 1 mm. As shown in Figure 5, the diffusion current increases with an increasing concentration ratio due to the corresponding increase in the diffusion potential. However, a limiting effect occurs as the concentration ratio is increased beyond 1000:1 (mM). Similarly, Figure 6 shows that the transference number increases (i.e., the Nafion membrane becomes increasingly cation selective) as the concentration ratio is increased from 10:1 to 1000:1 (mM), but reduces as the concentration ratio is further increased to 2000:1 (mM). 

For an RED system, the energy conversion efficiency is defined as the ratio of the output electrical energy to the input Gibbs free energy of mixing. Moreover, the efficiency obtained under the maximum power condition, η_max, power_, can be expressed as [21,27]:

(6) $$\eta_{\max,\;\mathrm{power}}\;=\;\frac{{\left(2t_{+}\;-\;1\right)}^{2}}{2}$$

**Figure 7:**
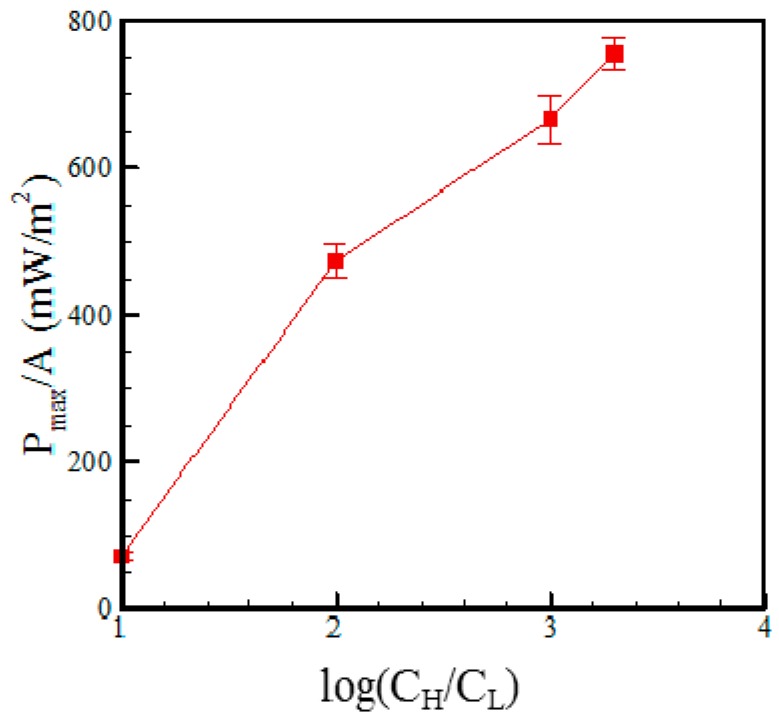
Variation of the maximum power density with concentration ratio given a microchannel length of 1 mm.

**Figure 8:**
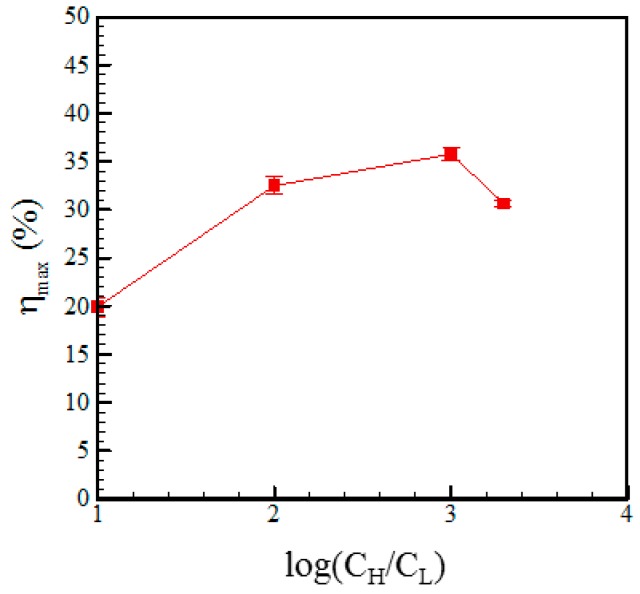
Variation of the energy conversion efficiency with the concentration ratio given a microchannel length of 1 mm.

**Figure 9:**
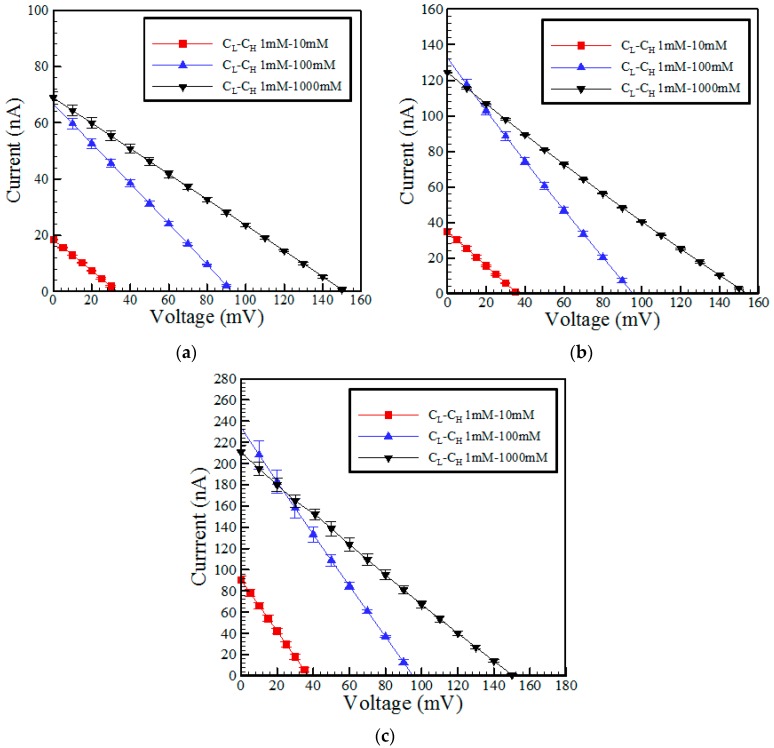
Current-potential curves for the different concentration ratios in RED devices with microchannels of lengths of: (**a**) 3 mm; (**b**) 2 mm; and (**c**) 1 mm.

Figure 7 shows the maximum power density (i.e., the maximum power divided by the membrane area) in the RED device with a microchannel length of 1 mm given concentration ratios of 10:1, 100:1, 1000:1 and 2000:1 (mM). It is seen that the maximum power density has a value of 755 mW/m^2^ and is achieved in the device with a concentration ratio of 2000:1 (mM). By contrast, the highest conversion efficiency is achieved in the device with a concentration gradient of 1000:1 (mM) and is found to have a value of 36%, as shown in Figure 8. Figure 9a–c show the *I-V* characteristics of the RED devices with channel lengths of 3, 2 and 1 mm, respectively, given three different electrolyte concentration ratios (10:1, 100:1 and 1000:1 (mM)) in each case. It is seen that for all values of the microchannel length, the short circuit current does not monotonically increase as the concentration ratio is increased. This finding can be explained as follows. Since cation migration flux from the high-concentration reservoir to the low-concentration reservoir increases with the concentration ratio, the amount of cations accumulated at the lower concentration reservoir would result in an opposite electrical field to the anode side (see Figure 3a). The opposite electrical field and the cation diffusion flux would therefore counteract and reach an equilibrium condition.

## 4. Conclusions

This study has fabricated a salinity-gradient RED device consisting of two microchambers connected by a straight Nafion-filled microchannel. The power generation performance of the proposed device has been evaluated for three different microchannel lengths (1, 2 and 3 mm) and four different KCl electrolyte concentration ratios (10:1, 100:1, 1000:1 and 2000:1 (mM)). The results have shown that the output current and power increase with a reducing microchannel length due to a lower channel resistance. For the RED device with a microchannel length of 1 mm, a maximum power density of 755 mW/m^2^ is achieved using a concentration ratio of 2000:1 (mM), while a maximum efficiency of 36% is obtained for a concentration ratio of 1000:1 (mM). Finally, the short circuit current does not monotonically increase as the concentration ratio is increased. The short circuit current is constrained at higher electrolyte concentration ratios since the higher electric field impedes the migration of the cations to the low-concentration reservoir.
